# Supplementary figures and images for: Using species richness calculations to model the global profile of unsampled pathogenic variants: Examples from BRCA1 and BRCA2
Source: PLoS One. 2023 Feb 8;18(2):e0278010. doi: 10.1371/journal.pone.0278010 (PMC9907816; doi:10.1371/journal.pone.0278010)

S1 Fig: Flow diagram of study search and selection procedure

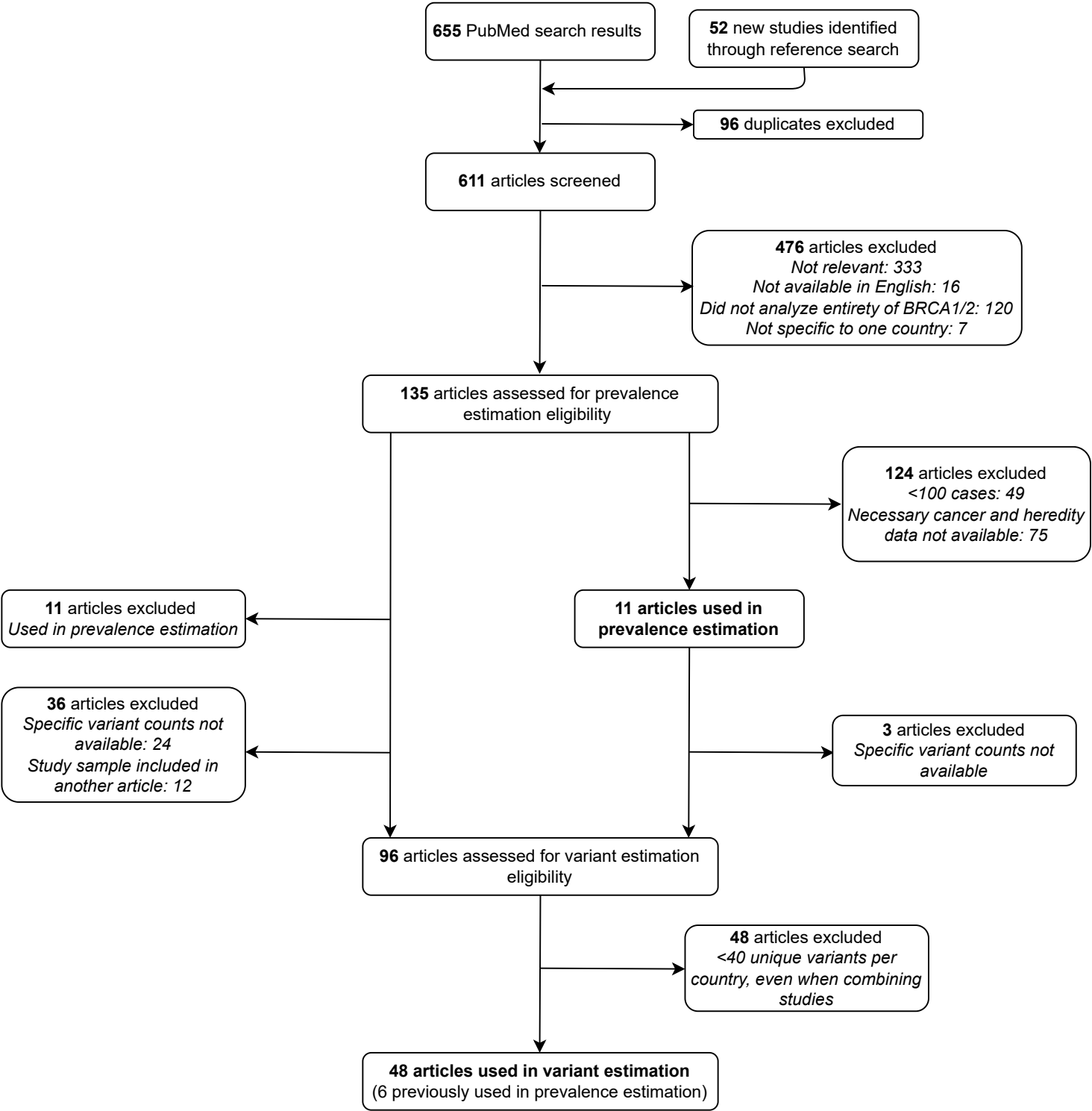

Supplement: S1 Fig — (PDF) [file pone.0278010.s001.pdf]
